# Surgery of the Appendix

**Published:** 1897-02

**Authors:** 


### SURGERY OF THE APPENDIX.

There is perhaps no topic at the present time which has greater interest to those who practice medicine and surgery than that of the surgical treatment of diseases of the vermiform appendix. This subject was considered in the Section of Surgery at the annual meeting at Carlisle, and a very interesting and important discussion took place. Every practitioner, as he stands by the bedside of a patient suffering from what he believes to be a severe attack of appendicitis, must have felt the grave responsibility of having to decide whether an operation should be performed. The question as to the desirability of early operation was the chief point under discussion, and all the surgeons who took part in it seemed to agree that early operation was most essential in many cases, and indeed gave the patient his only chance of recovery; and many warn, ings were uttered not to leave the operation until too late.

Several attempts were made to answer the very difficult question in what cases, and at exactly what time, we should operate. The practice of some American surgeons—Murphy of Chicago, and Morris, of New York—is to operate on all severe cases of appendicitis, for they consider that it is impossible to be sure that a case of what seems like appendicitis only may not be already one of general peritonitis from appendix infection, or that a collection of pus may not exist far earlier in the case than the examination of the abdomen can discover, and may rupture into the general peritoneal cavity and set up fatal peritonitis. Their statistics of more than two hundred cases of appendicitis—all of which were operated on—give a mortality distinctly lower than that of the disease under medical treatment; and they strongly maintain that the patient's safety lies in the isolation and removal of an appendix which may start fatal peritoneal infection at any moment. Mr. Morton's plea for operation in all severe cases was based on the experience of Murphy and Morris, and Dr. MacDougall and other speakers, although they did not advocate operation in all severe cases, went so far as to advise operation in some in the very early stage, before the mass of adherent bowel around the collection of pus could be adherent to the abdominal wall.

Most of the speakers advocated operation at an earlier period than that advised in one of the latest standard text-books of surgery in this country. On the other hand, some expressed a feeling almost of incredulity as to the results said to be obtained by American surgeons. Several cases, however, were related, or referred to, of patients lost because early operation had not been undertaken, and the effect of the discussion will doubtless be to stimulate practitioners to greater watchfulness for urgent symptoms in all cases of appendicitis, however trivial at the onset, and may perhaps lead some surgeons to give more consideration to this most important question, When should we operate in appendicitis?

Mr. Southam and Mr. Rutherford Morison agreed that operation should be undertaken in relapsing cases after the second attack, and attention was called by other speakers to the fact that a recurrent attack of appendicitis is sometimes fatal. This is not surprising if we remember that in several of the cases of relapsing appendicitis operated on by Mr. Treves the appendix was found full of pus, or custard-like material was found around it, which must have been the result of old abscess formation.

A subject which has recently been debated at an American surgical society by Professor White and Dr. McBurney and others, was also under discussion at Carlisle. We refer to the removal of the appendix in cases in which, on operation, pus is discovered and evacuated. Nearly every surgeon who took part in the discussion at Carlisle agreed with Dr. MacDougall that if the appendix could be readily found and easily removed it should be done, but that if it were necessary to risk the rupture of those adhesions around the pus, on which the safety of the general peritoneal cavity depends, it would be far better for the patient to leave the appendix alone. The American surgeons, in the debate referred to, arrived at the same conclusion.—*British Medical Journal.*

